# Zinc Porphyrin-Functionalized Fullerenes for the Sensitization of Titania as a Visible-Light Active Photocatalyst: River Waters and Wastewaters Remediation

**DOI:** 10.3390/molecules24061118

**Published:** 2019-03-21

**Authors:** Elzbieta Regulska, Danisha Maria Rivera-Nazario, Joanna Karpinska, Marta Eliza Plonska-Brzezinska, Luis Echegoyen

**Affiliations:** 1 Institute of Chemistry, University of Bialystok, Ciolkowskiego 1K, 15-245 Bialystok, Poland; joasia@uwb.edu.pl; 2 Department of Chemistry, University of Texas at El Paso, 500 W. University Ave., El Paso, TX 79968, USA; dmriveranazario@miners.utep.edu; 3 Department of Organic Chemistry, Faculty of Pharmacy with the Division of Laboratory Medicine, Medical University of Bialystok, Mickiewicza 2A, 15-222 Bialystok, Poland

**Keywords:** fullerene, photocatalyst, porphyrin, water remediation

## Abstract

Zinc porphyrin-functionalized fullerene [C60] derivatives have been synthesized and used to prepare titania-based composites. The electrochemical properties and HOMO and LUMO levels of the photosensitizers were determined by electrochemical measurements. Raman and IR techniques were used to study chemical groups present on the titania surface. Absorption properties of the composites were measured in the solid state by diffuse reflectance UV-Vis spectra (DRS). The zeta potential and aggregate sizes were determined using dynamic light scattering (DLS) and electrophoretic light scattering (ELS) techniques. Surface areas were estimated based on Brunauer–Emmett–Teller (BET) isotherms. The photocatalytic activity of the photocatalysts was tested using two model pollutants, phenol and methylene blue. The composite with the highest photocatalytic potential (1/TiO_2_) was used for river and wastewater remediation. The photodegradation intermediates were identified by LC-UV/Vis-MS/MS techniques.

## 1. Introduction

Since Fujishima and Honda [1] first demonstrated water splitting on titania (TiO_2_) electrodes, TiO_2_ has attracted considerable interest in the field of heterogeneous photocatalysis. The extensive usage of this semiconductor derives from its convenient properties like stability, chemical and biological inertness, no toxicity, low price, and environmental friendliness. Nevertheless, there are some significant drawbacks of titania, which preclude it from being used as an efficient photocatalyst. One limitation is the reasonably large energy band-gap of 3.2 eV, which requires ultraviolet light for its excitation, and another is the high probability for recombination of the photogenerated holes and electrons.

For that reason, the photosensitization of titania with different chromophores has been reported [2,3,4,5]. Artificial photosynthetic systems employing porphyrin-linked fullerene derivatives have been thoroughly examined in organic photovoltaic cells (OPVs). Porphyrins [6,7,8,9,10] and fullerenes [11,12,13,14,15,16,17,18,19] have also been independently employed for the preparation of modified titania. Nevertheless, to our knowledge, there are no reports involving covalently linked porphyrin–fullerene dyads as photosensitizers for titania photocatalysis. 

Heterogeneous photocatalysis is considered the most effective among the Advanced Oxidation Processes (AOPs), and has high potential for polluted water treatment. The development of effective treatment technologies for surfaces as well as for wastewaters is especially important in view of the increasing growth of water pollution by chemicals arising from many industrial, agricultural, and urban human activities. Photocatalytic treatment is considered the most promising technique due to its high efficiency, and is expected to replace other AOPs currently used for that purpose. 

**Figure 1:**
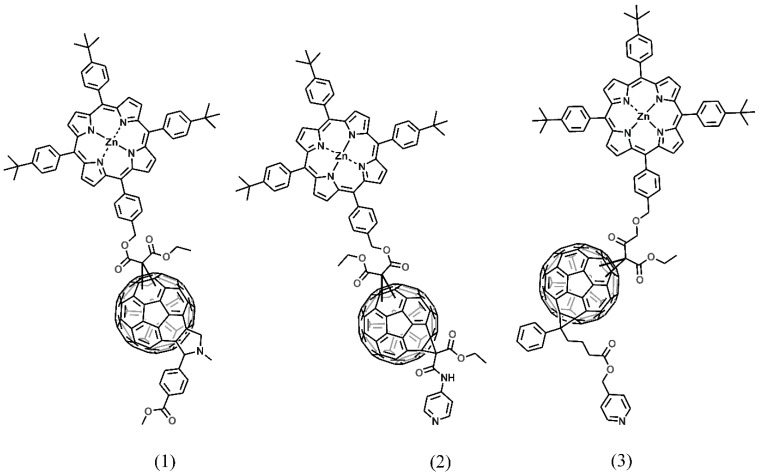
Structures of the fullerene derivatives: **1**, **2** and **3**.

In this article, we report the synthesis of three novel zinc porphyrin-functionalized fullerenes (see Figure 1), which were used to prepare titania-based composites and applied for the photocatalytic degradation of phenol and methylene blue as model pollutants. The photocatalyst exhibiting the highest activity was used for the remediation of selected river waters and water effluents.

## 2. Results and Discussion

### 2.1. Photoelectronic Properties of the Fullerene Derivatives 

**Figure 2:**
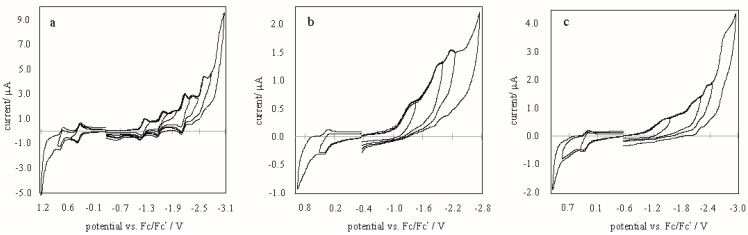
Cyclic voltammograms of **1** (**a**), **2** (**b**), **3** (**c**) in TBAPF_6_/(MeCN:Tol = 1:4, *v*/*v*), at a scan rate of 50 mV∙s^−1^.

The electrochemical characterization of the synthesized fullerene derivatives was performed to examine the relation between the energies of the LUMOs of the chromophores and the conduction band of titania, and to obtain the energy band gap of each compound. The CV cycles of the compounds are presented in Figure 2. 

Two waves due to the oxidation of the porphyrin macrocycle were observed in the anodic scan associated with the formation of P^•+^ and P^•2+^ [20]. Five, three, and four reduction waves were observed for the cathodic scan for **1**, **2** and **3**, respectively. They resulted from the reduction of porphyrin and fullerene, namely, two-electron reduction of the aromatic porphyrin ring overlapping with the reduction of the fullerene moiety. In terms of porphyrin, only the reduction of the ring is reflected on the CV curve, since the zinc(II) ion is electrochemically inactive [21]. Based on the onsets of the first oxidation and reduction peaks, the HOMO and LUMO levels of the compounds were estimated according to the following equations:

E_LUMO_ = −(4.71 − E_1/2, Fc,Fc+_ + E_red, onset_) (1)

E_HOMO_ = − (4.71 − E_1/2, Fc,Fc+_ + E_ox, onset_) (2)

**Figure 3:**
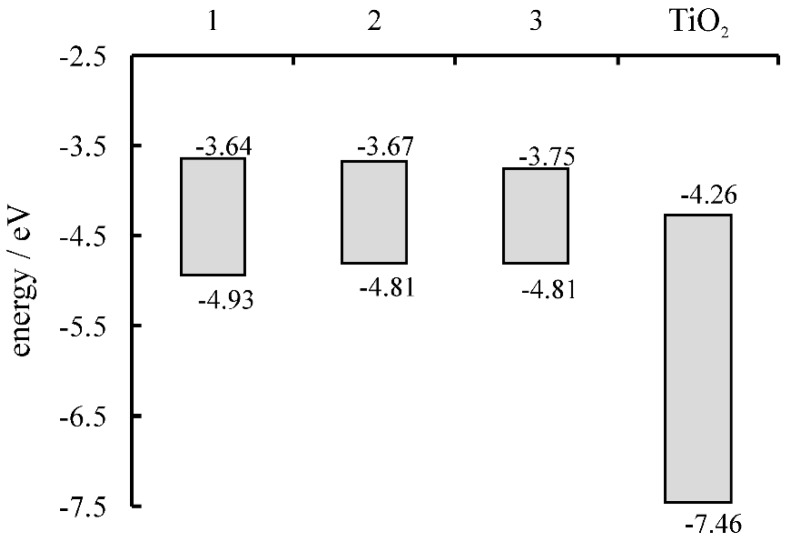
HOMO–LUMO energy levels for the synthesized fullerene derivatives (**1**–**3**) and TiO_2_.

The calculated HOMO and LUMO energy values of the fullerene derivatives are presented in Figure 3. The LUMOs of the photosensitizers are all above the energy of the conduction band (CB) of TiO_2_, and the differences are 0.62, 0.59, and 0.51 eV for **1**, **2**, and **3**, respectively. Therefore, efficient photosensitization of TiO_2_ by the fullerene derivatives should be observed. 

### 2.2. Characterization of the Titania 

**Figure 4:**
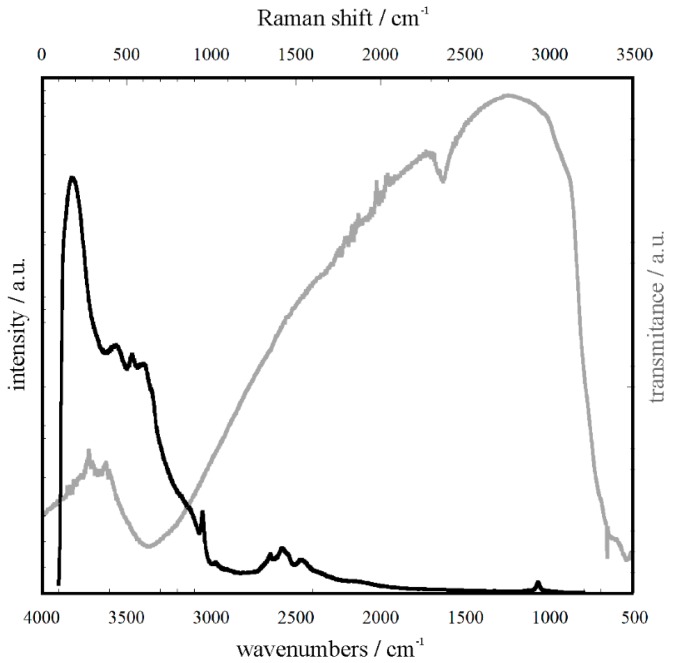
Raman (**black line**) and Attenuated Total Reflection-Fourier Transform Infra-Red (ATR-FTIR) (**grey line**) spectra of the synthesized titania.

Raman and IR spectra of the synthesized titania are shown in Figure 4. The Raman spectrum shows broad bands at the following Raman shifts: 100–260, 380–480, 500–650, and 800–900 cm^−1^, which were assigned to Ti–O bending, Ti–O stretching, and Ti–O–Ti stretching vibrations, respectively [22]. A band at 1020 cm^−1^ was assigned to the C–O group arising from the titanium precursor [22]. Typical bands arising from crystalline titania were not observed. Thus, the TiO_2_ prepared by the sol–gel synthesis was obtained in an amorphous phase. The IR spectrum (Figure 4) shows the presence of the hydroxyl groups on the oxide surface. The broad band observed in the range of 2500–3500 cm^−1^ results from O–H stretching vibrations [23]. Additionally, a band around 1710 cm^−1^ was assigned to the H–OH and Ti–OH bending vibrations [8,24], while the signals coming from the O–H in-plane and out-of-plane bending vibrations were observed at 410 cm^−1^ and in the range of 700–800 cm^−1^, respectively. The presence of the hydroxyl groups on the catalyst surface is crucial for its photocatalytic performance. Therefore, the prepared oxides, despite their amorphous structure, were expected to exhibit photocatalytic activity. 

### 2.3. Characterization of the Composites

**Figure 5:**
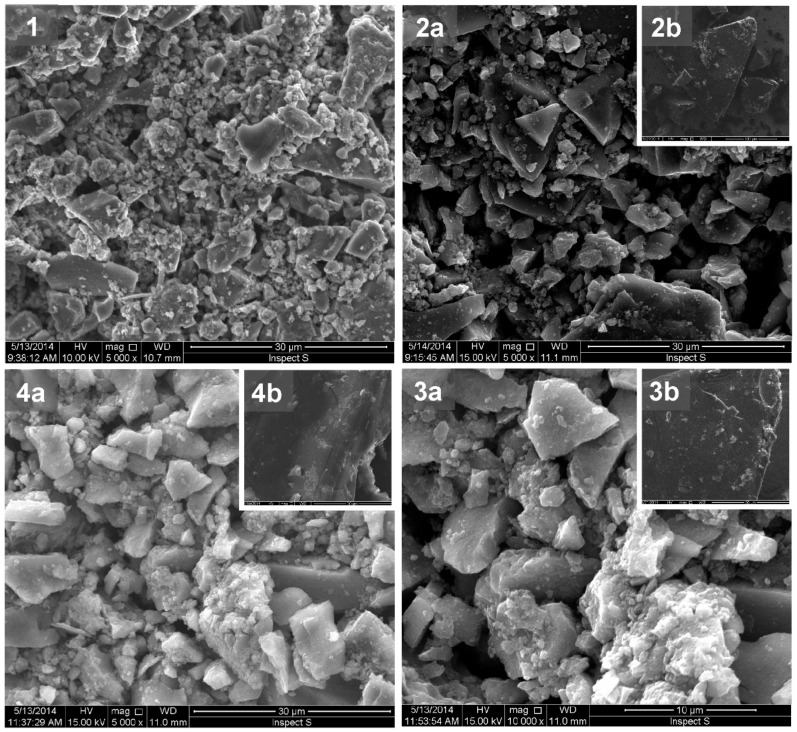
SEM images of titania (**1**), the composites TiO_2_/**1** (**2a**), TiO_2_/**2** (**3a**), and TiO_2_/**3** (**4a**) and the fullerene derivates **1** (**2b**), **2** (**3b**), and **3** (**4b**).

The morphologies of the composites were examined by SEM microscopy and compared with the respective components, i.e., titania and fullerene derivatives **1**, **2**, and **3** (see Figure 5). The pristine titania presented a porous structure (Figure 5-1), while SEM images of fullerene derivatives showed their smooth surfaces (Figure 5–2b,3b,4b), as we have previously reported for fullerenes [25,26]. The composites TiO_2_/**1**, TiO_2_/**2**, and TiO_2_/**3** were found to form irregular bricks covered with the porous titania (Figure 5–2a,3a,4a). The introduction of the smooth fullerenes affected the specific surface areas of the composites, since the BET areas of the composites were smaller than that of the pristine titania (see Table 1). 

**Figure 6:**
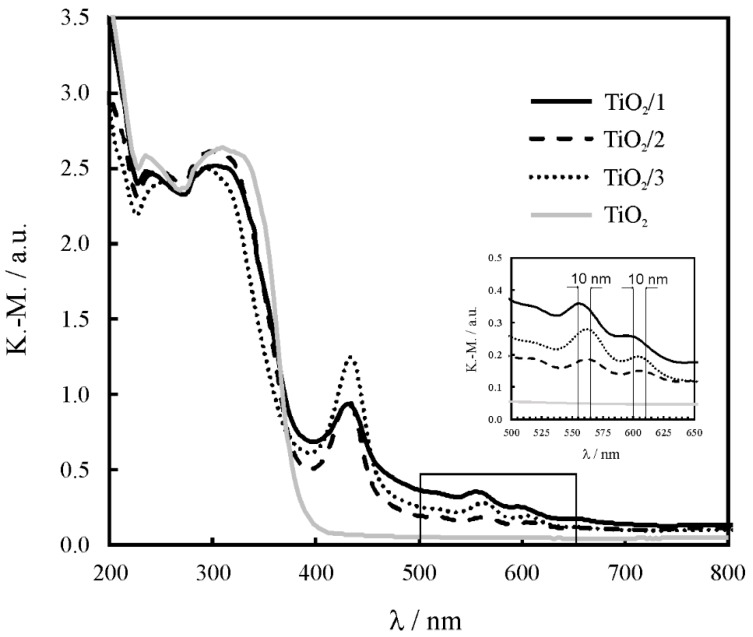
Kubelka–Munk plots of the titania-based composites with the fullerene derivatives: **1**, **2** and **3**.

The absorption spectra of the composites were compared with the spectrum of the pristine titania (Figure 6). The Soret and Q bands from the porphyrin macrocycles were observed for TiO_2_/**1**, TiO_2_/**2**, and TiO_2_/**3**. They appeared within the following wavelength ranges: 390–480 and 530–680 nm for Soret and Q bands, respectively. The fullerene absorption band was not observed due to overlap with the strong absorption of the titania in the range of 200–380 nm. The highest intensity of the Soret band was observed for TiO_2_/**3**, with a maximum at 420 nm. Moreover, a red-shift of the Q band was noticed for TiO_2_/**2** and TiO_2_/**3** when compared with the spectrum of TiO_2_/**1**. Therefore, TiO_2_/**3** exhibits the most efficient absorption in the visible spectral range. 

The specific surface areas of the titania and the corresponding composites were determined from BET isotherms. The results (Table 1) for the composites were in the range of 151–178 m^2^∙g^−1^, while the surface area of the pristine semiconductor was 269 m^2^∙g^−1^. Thus, the introduction of the fullerene derivatives in the composites resulted in decreases of the BET surface areas, as expected. 

The aggregate sizes of the TiO_2_ and the composites were determined by dynamic light scattering (DLS) measurements (see Table 1). The sizes of the catalyst aggregates may influence the kinetics of the photocatalytic degradation. It was previously reported that upon increasing the titania diameter from 0.35 to 0.66 μm, the photocatalytic activity of the semiconductor decreased by around 4.6 times [27]. In the present study, it was found that the diameters of the composite aggregates were 3 to 7 times lower when compared to that of the pristine titania. 

Additionally, electrophoretic light scattering (ELS) studies were performed to examine the dependence of the electrophoretic potential and the pH, in order to establish the range within which the catalysts form stable suspensions. The electric charge of the oxides in the solid state results from the presence of hydroxyl groups on their surface. Their amphoteric character results from their ability to react with both hydrogen and hydroxyl groups [27]. Bahnemann et al. [28] proposed the following notation for the chemisorption processes occurring on the surface of titania:

$$\mathrm{Ti}O_{2}+nH^{+}⇄\mathrm{Ti}O_{2}H_{n}^{n+}$$

$$\mathrm{Ti}O_{2}+nOH^{-}⇄\mathrm{Ti}O_{2}{\left(OH\right)}_{n}^{n-}$$

**Figure 7:**
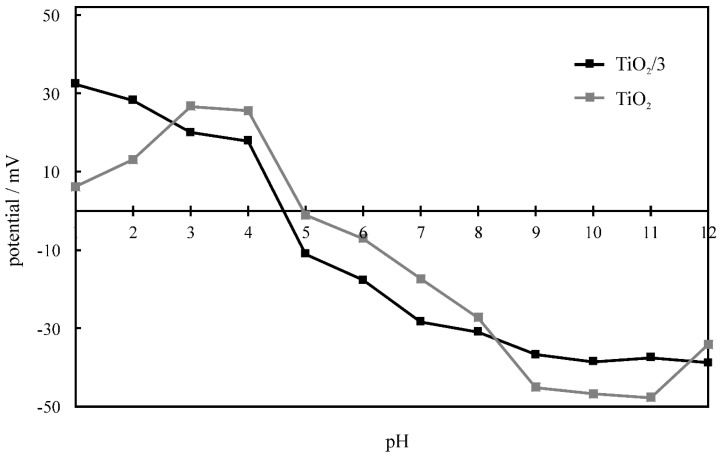
The efficiencies of phenol and methylene blue photocatalytic degradation in the presence of TiO_2_ and the given composites.

The high repulsion force between particles of the same charge decreases the probability of aggregation, thus increasing the stability of the sols in acidic and basic environments. The relation between the zeta potentials and the pH for TiO_2_ and TiO_2_/**3**, is shown in Figure 7. In both cases, a similar dependence was observed. Zero-point potentials appeared at pH levels equal to 4.9 and 5.6 for TiO_2_ and TiO_2_/**3**, respectively. For TiO_2_, in the pH range of 1.0–4.0, a 20 mV increase of the potential was observed. The decrease of the zeta potential continued until the potential reached −45 mV at pH 9.0. Stable suspensions with TiO_2_ are expected to be formed only between pH values of 8 and 12 pH, while within that range, absolute values of zeta potentials exceeded 30 mV. For TiO_2_/**3**, the zeta potential decreased with the increasing pH over the complete pH range. The absolute value of the zeta potential was higher than 30 mV within the pH range of 7–12. Therefore, under these conditions, the highest stability expected is for the TiO_2_/**3** aggregate. The composite particles may tend to aggregate and not be able to form stable suspensions within the pH range of 3–7.

### 2.4. Photocatalytic Performance

**Figure 8:**
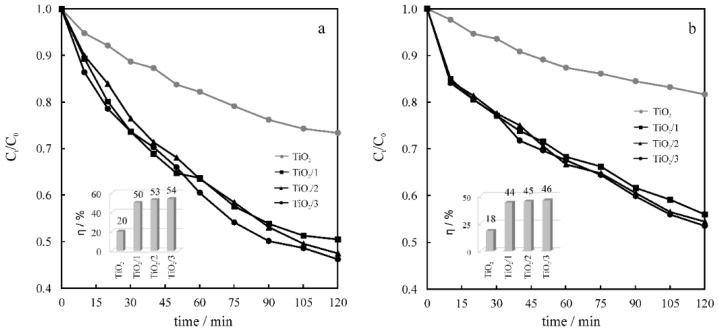
The decrease of phenol (**a**) and methylene blue (**b**) relative concentrations, and the efficiencies (insets) of their photocatalytic degradation in the presence of TiO_2_ and the given composites.

The photocatalytic performance of the composites was tested using two model pollutants, phenol (PhOH) and methylene blue (MB). The decreases of their relative concentrations and the efficiencies (η) of their photocatalytic degradation in the presence of the catalysts are presented in Figure 8. 

**Figure 9:**
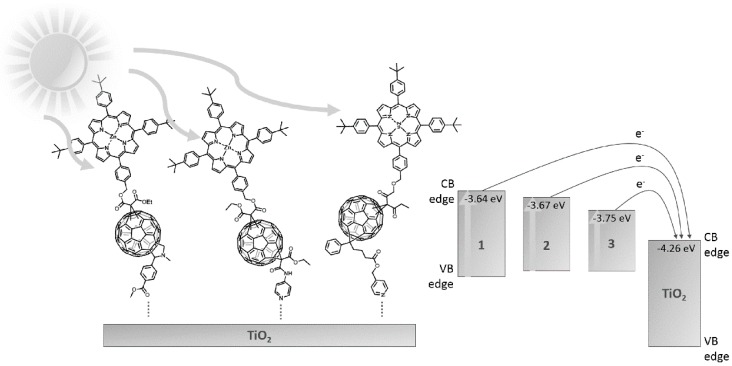
The proposed mechanism of the photocatalytic activity of the porphyrin-functionalized fullerenes/titania composites.

The highest η during the degradation of PhOH was achieved using TiO_2_/**3** (54%). However, similar values were also obtained with TiO_2_/**1** (50%) and TiO_2_/**2** (53%). Introduction of **3** as a photosensitizer enhanced the photocatalytic activity of TiO_2_ by nearly three times (2.7). When MB was exposed to the solar-simulated light in the presence of the composites, the degradation obtained was between 44% and 46%. Again, the highest η was observed for TiO_2_/**3**, at 2.5 times compared with pristine TiO_2_. Slightly lower η values were observed when MB was exposed to the radiation in the presence of the photocatalysts. This was attributed to the competitive light absorption between the catalyst and the model compound. The highest photocatalytic activity was shown by TiO_2_/**3** when both model pollutants were used. The proposed mechanism explaining the enhancement of the photocatalytic activity through the formation of the porphyrin-functionalized fullerene/titania composites versus pristine titania is presented in Figure 9. The improved performance of the synthesized catalysts derives from the utilization of the solar light for the excitation of the fullerene derivatives. We attribute the observed highest activity of TiO_2_/**3** to both the smallest difference between the LUMO of **3** and the CB of TiO_2_, and to the smallest energy band-gap of **3** among the photosensitizers. 

### 2.5. UV–Vis Absorbance Spectra of the Photodegradation Products 

**Figure 10:**
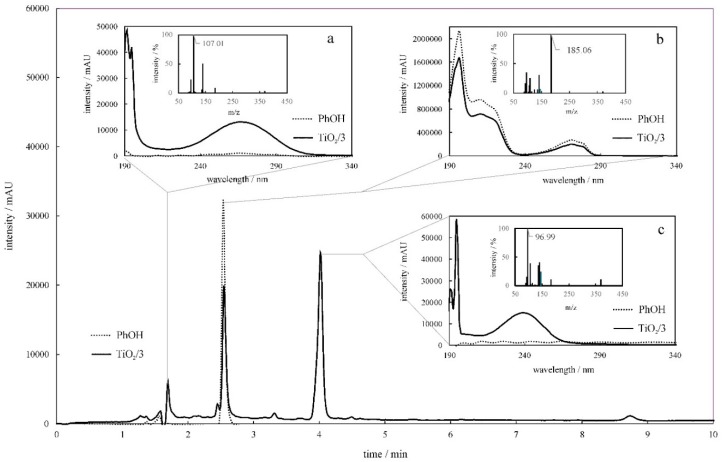
The chromatograms of phenol (PhOH) initial solution (**dotted line**) and after 2 h of photocatalytic treatment (**solid line**) with TiO_2_/**3**, recorded at 239 nm. Absorption spectra corresponding to the peaks are registered in the chromatograms at 1.71 (**a**), 2.56 (**b**), and 4.05 min (**c**). Insets represents mass spectra registered at respective retention times.

The photodegradation products of PhOH and MB during their exposure to solar light in the presence of TiO_2_/**3** were examined using the LC-UV–Vis-MS technique. Figure 10 depicts a chromatogram, registered after 2 h of PhOH irradiation, that shows three peaks. Based on the UV–Vis absorption and mass spectra registered at 1.71, 2.56, and 4.05 min, the main photodegradation products were found. They were identified as 4,4’-dihydroxybiphenyl, benzoquinone, and maleic anhydride. It was also shown that under the applied conditions, complete phenol decomposition was not accomplished. Additional intermediates were identified by mass spectrometry in minor amounts (see Supporting Information). 

**Figure 11:**
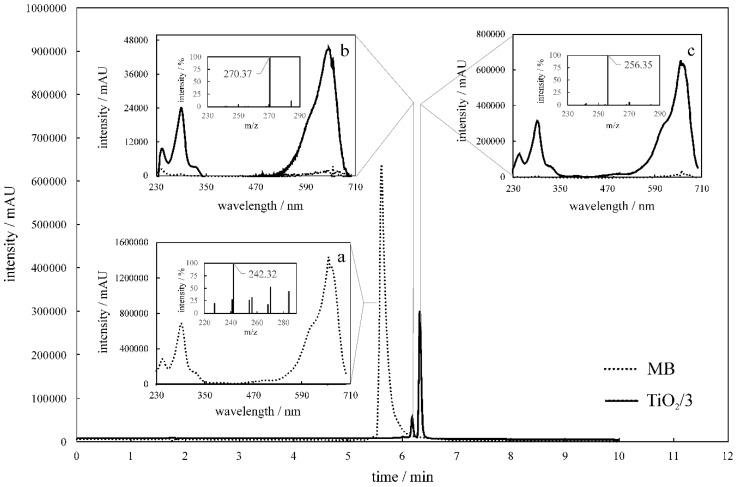
The chromatogram of the initial methylene blue (MB) solution (**dotted line**) and after 2 h of photocatalytic treatment (**solid line**) with TiO_2_/**3** recorded at 610 nm. Absorption spectra corresponding to the peaks are registered in the chromatogram at 5.61 (**a**), 6.20 (**b**), and 6.34 min (**c**). Insets are the mass spectra registered at the respective retention times.

Contrary to PhOH, MB was completely degraded under the conditions used. In Figure 11, chromatograms of MB before and after its irradiation in the presence of TiO_2_/**3** are presented, along with the absorption spectra (Figure 11a–c) corresponding to the retention times at which pristine MB and its photodegradation intermediates were observed. The peak arising from MB did not appear in the chromatogram obtained after 2 h of photocatalytic treatment. However, two new peaks at 6.20 and 6.34 min were observed. Based on their absorption spectra (Figure 11b,c), it was found that the MB photodegradation intermediates absorb visible light. Even though MB was not completely decolorized, it is likely that optimal adjustment of irradiation times and higher light intensity can accomplish this in the future. A list of the identified MB photodegradation products is included in the Supporting Information. 

### 2.6. Photocatalytic Performance Using Natural Samples

**Figure 12:**
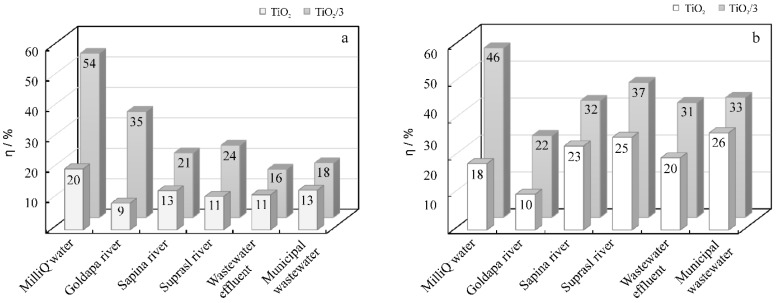
Efficiencies (η) of PhOH (**a**) and MB (**b**) photocatalytic degradation in the presence of TiO_2_ or TiO_2_/**3**, and the following matrices: MilliQ water, Goldapa, Sapina, and Suprasl river waters, wastewater effluent, and municipal wastewater.

In order to understand the complex nature of environmental matrices, the contribution of different ions, as well as of organic compounds, needs to be considered. A series of natural matrices, including three river waters, wastewater effluent, and municipal wastewater, were used in our studies. The efficiencies of PhOH and MB photocatalytic degradation in the presence of TiO_2_ or TiO_2_/**3** are presented in Figure 12. Photocatalytic degradation conducted with the natural matrix was found to be significantly slowed down compared with the one performed in the MilliQ water. This behavior is due to the complex composition of natural samples, which leads to lower light absorption efficiencies due to competition for access to light between the catalyst and the natural water ingredients. In addition, the radicals formed during the photocatalytic reactions may react with a wide variety of substances present in the matrix, rather than resulting in the direct decomposition of the model pollutant. In this regard, the following parameters are particularly important: the oxygen content, the presence of transition metal cations, the concentration of ions responsible for water hardness, dissolved organic carbon, and the color of the sample [29]. A series of parameters was determined for both the examined river waters and wastewaters, which are presented in Table 2 and Table 3, respectively. The estimated values enabled the assignment of the examined river waters to the second grade of quality [30], and for the wastewater effluents to meet the expected criteria for effluents.

Different river waters affected the decomposition of the model pollutants differently. When PhOH was subjected to photocatalytic degradation with TiO_2_/**3**, the smallest decrease of η was observed in Goldapa river water (35%) when compared to the η obtained in MilliQ water (54%). However, MB decomposition was found to decrease the least in Suprasl river water (from 46% to 37%). We found that the series of parameters, including concentration of chlorine, calcium, magnesium, and nitrate ions, along with hardness and conductivity, were found to be the highest in Suprasl river waters. However, some of the species exhibit both positive and negative effects on photodegradation.

The total hardness of the water is a factor which negatively influences the performance of the photocatalytic processes. This parameter is related to the content of carbonate and bicarbonate ions, which were estimated to be the highest for the Suprasl river. These anions react with the hydroxyl radicals to generate other less reactive radical species according to the following chemical equations:

CO_3_^2−^ + HO^•^ → CO_3_^•−^ + HO^•−^

HCO_3_^2−^ + HO^•^ → CO_3_^•−^ + H_2_O

It is generally believed that metal cations which are present in only one oxidation state do not affect the photocatalytic decomposition either positively or negatively. However, it was shown that the process of photocatalytic degradation can be accelerated in the presence of calcium ions. Mariquit et al. [32] showed that calcium cations support the adsorption of humic acid, thus facilitating photocatalytic oxidation. Furthermore, Yiang et al. [33], while examining typical organic pollutant 1-methylimidazole-2-thiol, indicated a faster photocatalytic degradation of the compound after the introduction of calcium ions. This parameter was the highest in the Suprasl river as well.

On the other hand, oxygen participates in the processes described below, being a source of the oxygen radicals. It can be adsorbed on the catalyst’s surface and can undergo reactions with the participation of the conduction band of TiO_2_ [33]:

e^−^ + O_2_ → O_2_^−^ (ad)

O_2_^−^ (ad) + H^+^ → HO_2_^•^

The valence band of titania delivers other active forms of oxygen, like HO^•^ and ^•^O, which results from the presented equations:

h^+^ + H_2_O → HO^•^ (ad) + H^+^

h^+^ + O_2_^−^ (ad) → 2 ^•^O (ad)

All of the oxygen forms, like O_2_^−^, HO^•^, HO_2_^•^, and ^•^O, likely participate in the reactions leading to the degradation of the organic matter [34]. Dissolved oxygen was found to be the highest in the Sapina river, but this was not the matrix in which photodegradation was the most efficient.

The yield of the photocatalytic degradation in the presence of a river water sample may also be higher than in deionized water. Both rivers and wastewaters contain chromophores, which may also be involved in photochemical reactions leading to the formation of reactive compounds, which can initiate the decomposition of the model pollutants [35]. This was observed when MB solutions prepared in both rivers and wastewaters were irradiated in the presence of TiO_2_ (Figure 12b). It is believed that the presence of colored dissolved organic matter (CDOM) is responsible for the formation of singlet oxygen ^1^O_2_ and triplet excited states ^3^CDOM*. These forms may be even more important than the hydroxyl radical HO^•^ in the photodegradation processes. CDOM exposed to sunlight undergoes the following reactions [36]:

CDOM + hv → ^1^CDOM^*^

^1^CDOM^*^ → ^3^CDOM^*^

^3^CDOM^*^ → CDOM

^3^CDOM^*^ + O_2_ → CDOM + O_2_

^3^CDOM^*^ + O_2_ → CDOM + ^1^O_2_

^3^CDOM^*^ + P → CDOM^•−^ + P^•+^

CDOM^•−^ + O_2_ → CDOM + O_2_^•+^

^1^O_2_ → O_2_

^1^O_2_ + P → P_ox_

where *P* is phenol or its derivatives.

Photocatalytic degradations in environmental matrices are very complex processes. It is really difficult, if not impossible, to predict the influence of all of the ingredients of a natural matrix on the photodegradation yields. In the presented studies, the same photodegradation products of both model compounds were identified using MilliQ water and all of the environmental matrices as reaction mediums (Supporting Information).

## 3. Experimental

### 3.1. Methods

^1^H-NMR spectra were recorded on a JEOL ECA 600 NMR spectrometer (JEOL Ltd., Peabody, MA, USA) at room temperature using CDCl_3_ or CDCl_3_:CS_2_ as solvents. Mass spectra were obtained using a Bruker microFlex MALDI-TOF LRF spectrometer (Billerica, MA, USA) on reflector positive mode, using 1,8,9-trihydroxyanthracene or *trans*-2-[3-(4-*tert*-Butylphenyl)-2-methyl-2-propenylidene]malononitrile as the matrix. 

The Attenuated Total Reflection-Fourier Transform Infra-Red (ATR-FTIR) (Thermo Scientific, Waltham, MA, USA) spectra (500–3200 cm^−1^) were obtained using a Nicolet Model 6700 FT-IR spectrometer with a DTGS detector. The crystal-diamond spectra were obtained with 4 cm^−1^ resolution, and 32 scans for each sample spectrum were obtained. Diffuse reflectance UV–Vis spectra (DRS) were recorded on a Jasco V-30 UV-Vis/NIR spectrophotometer equipped with an integrating sphere 60 mm in diameter, using BaSO_4_ as a reference.

Raman spectra were collected using the Renishaw Raman InVia (Renishaw, New Mills, UK) Microscope equipped with a high sensitivity ultra-low noise CCD detector. The radiation from a high-power diode laser (Renishaw, New Mills, UK) (785 nm) was used as the excitation source. The laser power and accumulation of scans were dependent upon the sample. Spectra were recorded using ×50 objective with 10 s exposure time. The instrument was calibrated using an internal silicon standard (521 cm^−1^).

The sizes of the composite aggregates and zeta potentials were assessed using a NanoPlus-3 zeta/nano particle analyzer (Micromeritics Instrument Corporation, Norcross, GA, USA) for aqueous solutions. The sizes of the aggregates was determined based on dynamic light scattering (DLS) measurements for solutions of pH 7, while the zeta potentials were determined based on electrophoretic light scattering (ELS) measurements for solutions within pH range 1–14. The specific surface areas were measured using low-temperature nitrogen adsorption (80 K) with an ASAP 2020 (Micromeritics Instrument Corporation, Norcross, GA, USA) instrument. Prior to the measurements, samples were out-gassed under vacuum at 393 K for 12 h. The specific surface areas were determined using the Brunauer–Emmett–Teller (BET) method. 

Electrochemical measurements were performed in a mixture of MeCN:Tol = 1:4 (*v*/*v*). Tetrabutylammonium hexafluorophosphate (TBAPF_6_) was added as the supporting electrolyte after recrystallization from ethanol. Cyclic voltammetry (CV) experiments were performed under an argon atmosphere at room temperature, using a CH Instrument potentiostat (CHI-440B) (Metrohm AG, Herisau, Switzerland) at a scan rate of 100 mV∙s^−1^. A standard three-electrode set up, consisting of a glassy carbon working electrode (ALS Co., Ltd., 1 mm ID), a platinum wire (1.0 mm, Aldrich) as a counter electrode, and a silver wire (1.0 mm, Aldrich) as a pseudoreference electrode, was used. The redox couple of ferrocene/ferrocenium (Fc/Fc^+^) was used as an internal reference to measure the potentials.

The photocatalytic degradation experiments were investigated in a 50 mL glass cell. The reaction mixture consisted of 20 mL of the model pollutant (phenol (PhOH, 10^−4^ mol·L^−1^) or methylene blue (MB, 8 × 10^−5^ mol·L^−1^), aqueous solution, and a photocatalyst (1.5 g·L^−1^). Before the PhOH/MB photodegradation studies, adsorption experiments were carried out. The suspension of the model pollutant and the appropriate photocatalyst was kept in the dark while stirring for 1 h to attain equilibrium. To monitor the progress of the degradation during the irradiation (2 h), samples were taken and centrifuged every 15 min, and the supernatant was analyzed using UV–Vis spectroscopy. The UV–Vis spectra were recorded with a HITACHI U-2800A UV–Vis spectrophotometer equipped with a double monochromator and a single beam optical system (190–700 nm). A SUNTEST CPS+ (ATLAS, USA) solar simulator apparatus, emitting irradiation in the range of 380–700 nm, was used to perform photocatalytic degradation experiments. All experiments were repeated three times, and the presented results reflect the averaged data. The photon flux of solar-simulated radiation was equal to 2.32 × 10^−6^ Einstein s^−1^ for 250 W m^−2^. Identification of the model pollutant photodegradation products was performed using a Shimadzu Nexera ultra high-performance liquid chromatograph with a photodiode-array detector (Shimadzu Corporation, Kyoto, Japan) and a triple quadrupole mass spectrometer (Shimadzu LCMS-8040) (Shimadzu Corporation, Kyoto, Japan) equipped with an electrospray ionization (ESI) interface. 

### 3.2. Synthesis of Fullerene Derivatives

Fullerene derivatives **1**, **2**, and **3** were synthesized via a Bingel–Hirsch reaction. General procedure: 1 eqv. of the porphyrin malonate **8** (see Supplementary Materials) was reacted with 1.2 eqv. of the fullerene C_60_ derivative (**9**, **13**, or **14**; see Supporting Information) in the presence of CBr_4_ (1.1 eqv.) and upon addition of 1,8-diazabicyclo[5.4.0]undec-7-ene (DBU) (1.2 eqv.). The reaction was completed within 3 h. Compounds **1**, **2**, and **3** were isolated through the column chromatography as a mixture of isomers. For detailed procedures, see Supporting Information.

### 3.3. Synthesis of Titania

2 mL of titanium isopropoxide (TIPT) was dissolved in 5 mL of anhydrous ethanol with 0.5 mL of glacial acetic acid. After 30 min of stirring, the prepared solution was added dropwise to 5 mL of 5% sodium dodecyl sulfate (SDS) solution. The obtained mixture was left for 72 h under stirring. The prepared gel was washed 3 times with ethanol and water, whereupon it was evaporated under reduced pressure. The obtained powder was dried by placing in the oven (120 °C) for 24 h.

### 3.4. Synthesis of Composites

0.018 g of **1**, **2**, or **3** were placed in a round-bottom flask with 5 mL of CHCl_3_ and 5 mL of 5% aqueous SDS solution. The obtained mixture was sonicated for 12 h (solution A). Independently, an ethanolic (20 mL) solution of TIPT (666 μL) and glacial acetic acid (1 mL) (solution B) was prepared. Solution B was left under stirring for 30 min, whereupon it was added dropwise to solution A. The mixture of A and B was placed in a round-bottom flask and stirred for 2 h. The obtained gel was washed 3 times with ethanol and water, whereupon it was evaporated under reduced pressure. The obtained powder was dried by placing in the oven (120 °C) for 24 h.

## 4. Conclusions

Three novel fullerene derivatives covalently linked to a zinc porphyrin were synthesized and applied for the sensitization of titania. This paper represents the first report of the application of porphyrin-functionalized fullerene/titania composites as photocatalysts. It was found that the LUMO levels of the modified fullerenes are appropriate for proper electron injection into the conduction band of titania. Compound **3**, which exhibits the smallest energy difference with respect to the CB of TiO_2_, was the most active catalyst. The photocatalytic activities of the composites were tested against MB and PhOH in aqueous solutions as well as in a series of environmental matrices. The photocatalytic activities of all of the composites were comparable. Nevertheless, the highest photodegradation efficiency was observed for TiO_2_/**3**. It was shown that it presents photocatalytic activity in all of the examined river waters, along with municipal wastewater and wastewater effluents. It was observed that it can form stable suspensions in solutions over a pH range of 7–12. Therefore, it can be used for both river and wastewater remediation, since rivers and wastewaters show neutral and basic pH levels, respectively. It should also be stressed that both colorless and colored substances, like PhOH and MB, undergo degradation in the presence of the composites. Therefore, it can be assumed that they could be used not only for remediation of rivers and municipal wastewaters, but also of industrial wastewaters. In particular, the textile industry releases significant amounts of dyes, which are difficult to remediate. Accordingly, we believe that the results presented here show potential applications for the decontamination of a series of complex environmental and industrial matrices, using solar light as an irradiation source.

## Figures and Tables

**Table 1:** Brunauer–Emmett–Teller (BET) surface areas and diameters determined for titania and titania-based composites.

Catalyst	TiO_2_	TiO_2_/1	TiO_2_/2	TiO_2_/3
BET surface area/m^2^∙g^−1^	269	178	151	158
Diameter/μm	3.24	1.07	0.86	0.48

**Table 2:** River water parameters.

Parametr	Goldapa River	Sapina River	Suprasl River	The Range of Acceptable Values for Rivers [30]	Norms
pH	7.8	7.8	7.5	6.5-8.5	PN-90/C-04540/01
Acidity/mg CaCO_3_ ∙ L^−1^	111.6	99.2	80.6	nd	PN-90/C-04540/03
Alkalinity/mg CaCO_3_ ∙ L^−1^	240.2	260.2	180.2	≤150 (I) *; ≤250 (II) **	PN-90/C-04540/03
COD ***/mg O_2_ ∙ L^−1^	1.9	2.3	2.3	≤6 (I); ≤12 (II)	PN-85/C-04578/02
Conductivity/S ∙ cm^−1^	245	270	360	≤1000 (I); ≤1500 (II)	
Chlorine (Cl^−^)/mg Cl^−^ ∙ L^−1^	156.0	113.4	170.2	≤200 (I); ≤300 (II)	PN-75/C-04617/02
Calcium (Ca^2+^)/ mg Ca^2+^ ∙ L^−1^	56.1	74.1	104.2	≤100 (I); ≤200 (II)	PN-91/C-04551/01
Magnesium (Mg^2+^)/ mg Mg^2+^ ∙ L^−1^	27.3	16.0	35.3	≤50 (I); ≤100 (II)	PN-71/C-04554/10
Hardness/mg CaCO_3_ ∙ L^−1^	207.6	223.6	347.3	≤300 (I); ≤500 (II)	PN-71/C-04554/02
Dissolved oxygen/mg O_2_ ∙ L^−1^	0.9	2.2	1.0	≥7 (I); ≥5 (II)	ISO 5813:1983
Nitrate (V)/mg ∙ L^−1^	3.7	3.4	69.3	≤2.2 (I); ≤5.0 (II)	PN-82/C-04576/08
Phosphate/mg ∙ L^−1^	11.5	7.3	nd	≤0.20 (I); 0.31 (II)	PN-88/C-04537/04

* First grade of quality, ** Second grade of quality, *** Chemical oxygen demand.

**Table 3:** Municipal wastewater and wastewater effluents parameters.

Parameter	Municipal Wastewater	Wastewater Effluents	The range of Acceptable Values for Effluents [31]	Norms
pH	10.8	10.7	6.5-9.0	PN-90/C-04540/01
Acidity/mg CaCO_3_ ∙ L^−1^	2.3	1.1	nd	PN-90/C-04540/03
Alkalinity/mg CaCO_3_ ∙ L^−1^	580.0	285.0	150-350	PN-90/C-04540/03
COD/mg O_2_ ∙ L^−1^	89.8	36.7	<125	PN-85/C-04578/02
Conductivity/S ∙ cm^−1^	8500	7000	nd	
Chlorine (Cl^−^)/mg Cl^−^ ∙ L^−1^	368.7	326.1	<1000	PN-75/C-04617/02
Calcium (Ca^2+^)/mg Ca^2+^ ∙ L^−1^	78.6	86.7	nd	PN-91/C-04551/01
Magnesium (Mg^2+^)/mg Mg^2+^ ∙ L^−1^	41.7	17.6	>300	PN-71/C-04554/10
Hardness/mg CaCO_3_ ∙ L^−1^	300.2	260.2	nd	PN-71/C-04554/02
Dissolved oxygen/mg O_2_ ∙ L^−1^	nd	1.4	nd	ISO 5813:1983
Nitrate (V)/mg ∙ L^−1^	1226.3	45.0	<30	PN-82/C-04576/08
Phosphate/mg ∙ L^−1^	3418.5	18.1	nd	PN-88/C-04537/04

## Supplementary Materials

The following are available online at https://www.mdpi.com/1420-3049/24/6/1118/s1, Figure S1: Synthetic route to porphyrin (**8**): **(i)** MeOH, HCl, 65 °C, **(ii)** pyrrole, 4-*tert*-butyl benzaldehyde, TFA, DDQ, CHCl_3_, **(iii)** Zn(OA)_2_, CHCl_3_, **(iv)** LiALH_4_, THF, **(v)** TEA, ethyl malonyl chloride, THF, CH_2_Cl_2_; Figure S2: Synthetic route to fullerene (**1**): **(i)** (4), sarcosine, *o*-DCB, 180 °C, **(ii)** (**8**), DBU, CH_2_Cl_2_, Tol; Figure S3: Synthetic route to fullerene (**2**): **(i)** TEA, CH_2_Cl_2_, **(ii)** C_60_, CBr_4_, DBU, Tol, CH_2_Cl_2_, **(iii)** (**8**), DBU, CH_2_Cl_2_, Tol; Figure S4: Synthetic route to fullerene (**3**): **(i)** HCl, MeOH, 65 °C, **(ii)** TsNHNH_2_, MeOH, 65 °C, **(iii)** C_60_, NaOMe, pyridine, *o*-DCB, 180 °C, **(iv)** (**8**), DBU, CH_2_Cl_2_, Tol; Figure S5: PXRD images of the as-synthesized titania (a) and the annealed at 450 °C (b); Table S1: List of identified compounds obtained in the photocatalytic degradation of PhOH; Table S2: List of identified compounds obtained in the photocatalytic degradation of MB.
