# Supplementary material for: Zinc Porphyrin-Functionalized Fullerenes for the Sensitization of Titania as a Visible-Light Active Photocatalyst: River Waters and Wastewaters Remediation
Source: Molecules. 2019 Mar 21;24(6):1118. doi: 10.3390/molecules24061118 (PMC6471970; doi:10.3390/molecules24061118)
Supplement: Supplementary file 1 [file molecules-24-01118-s001.pdf]

Supplementary information

Zinc porphyrin functionalized fullerenes for the sensitization of titania as visible-light active photocatalyst: river waters and wastewaters remediation

Elzbieta Regulska [1,*], Danisha Maria Rivera-Nazario [2], Joanna Karpinska [1],
Marta Eliza Plonska-Brzezinska [3,*] and Luis Echegoyen [2,*]

[1] Institute of Chemistry, University of Bialystok, Ciolkowskiego 1K, 15-245 Bialystok, Poland. e.regulska@uwb.edu.pl
[2] Department of Chemistry, University of Texas at El Paso, 500 W. University Ave., El Paso, TX 79968, USA.
[3] Department of Organic Chemistry, Faculty of Pharmacy with the Division of Laboratory Medicine, Medical University of Bialystok, Mickiewicza 2A, 15-222 Bialystok, Poland

1. Materials

4-(aminomethyl)pyridine, buckminsterfullerene, cobaltous acetate dihydrate, copper acetate dihydrate, 1,8-Diazabicyclo[5.4.0]undec-7-ene (DBU), 2,3-dichloro-5,6-dicyano-1,4-benzoquinone (DDQ), *N,N′*-dicyclohexylcarbodiimide (DCCD), 4-(dimethylamino)pyridine (DMAP), *N,N′*-dimethylethanolamine (DMEA), ethyl acetate, ethyl malonyl chloride, lithium aluminium hydride ($LiAlH_4$), methyl 4-formylbenzoate, 5-oxo-5-phenylvaleric acid, phenol (PhOH), *p*-toluenesulfonyl hydrazide, pyridine, 4-pyridinemethanol, pyridinium chlorochromate (PCC), silica gel (230-400 mesh), sodium bicarbonate, sodium dodecyl sulfate (SDS), sodium methoxide, sodium sulphate, 4-*tert*-butylbenzaldehyde, tetrabromomethane, tetrabutylammonium hexafluoro-phosphate ($TBAPF_6$), titanium isopropoxide (TIPT), trifluoroacetic acid (TFA), trimethylamine (TEA), zinc acetate dihydrate were purchased from Sigma-Aldrich, hydrochloric acid solution from Fisher Scientific, while methylene blue from Park Scientific. All above mentioned chemicals were analytical grade reagents and used without further purification. HPLC-grade carbon disulfide (CS_2), chlorobenzene (PhCl), chloroform ($CHCl_3$), deuterated chloroform ($CDCl_3$), 1,2-dichlorobenzene (*o*-DCB), dichloromethane (DCM), ethyl acetate, hexane, methanol (MeOH), tetrahydrofuran (THF) and toluene (all Sigma-Aldrich), anhydrous ethanol (Merck) and glacial acetic acid (POCh) were used.

2. Synthesis

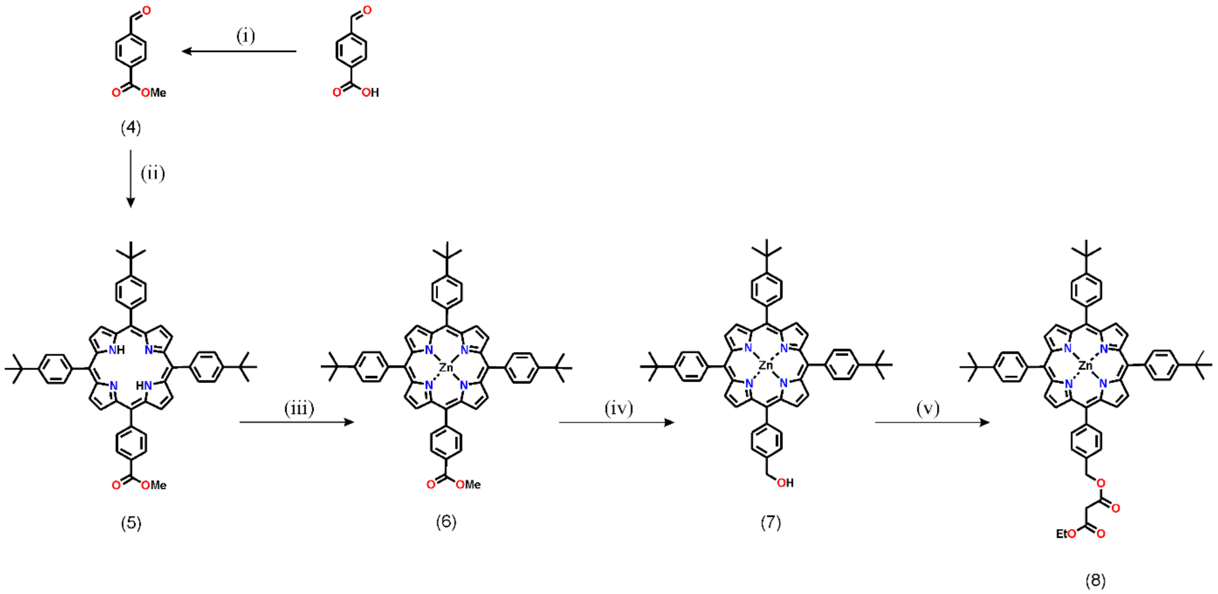

Figure S1. Synthetic route to porphyrin (**8**): **(i)** MeOH, HCl, 65°C, **(ii)** pyrrole, 4-*tert*-butyl benzaldehyde, TFA, DDQ, $CHCl_3$, **(iii)** $Zn(OA)_2$, $CHCl_3$, **(iv)** $LiALH_4$, THF, **(v)** TEA, ethyl malonyl chloride, THF, CH_2Cl_2.

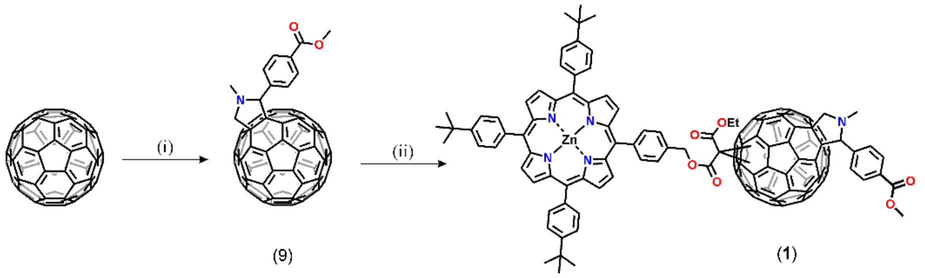

Figure S2. Synthetic route to fullerene (**1**): **(i)** (4), sarcosine, *o*-DCB, 180°C, **(ii)** (**8**), DBU, CH_2Cl_2, Tol.

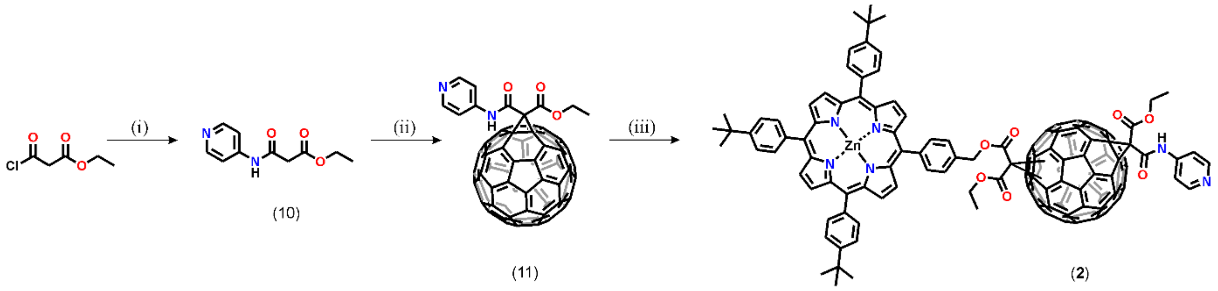

Figure S3. Synthetic route to fullerene (**2**): **(i)** TEA, CH_2Cl_2, **(ii)** C_{60}, CBr_4, DBU, Tol, CH_2Cl_2, **(iii)** (**8**), DBU, CH_2Cl_2, Tol.

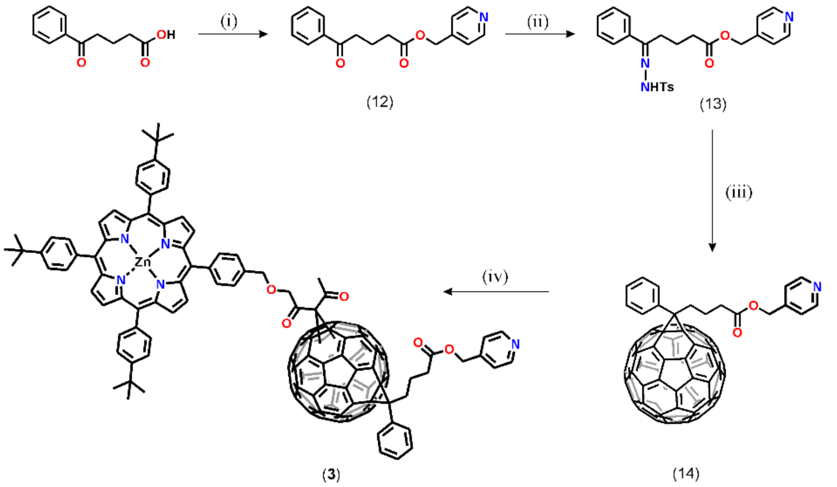

Figure S4. Synthetic route to fullerene (**3**): **(i)** HCl, MeOH, 65°C, **(ii)** $TsNHNH_2$, MeOH, 65°C, **(iii)** C_{60}, NaOMe, pyridine, *o*-DCB, 180°C, **(iv)** (**8**), DBU, CH_2Cl_2, Tol.

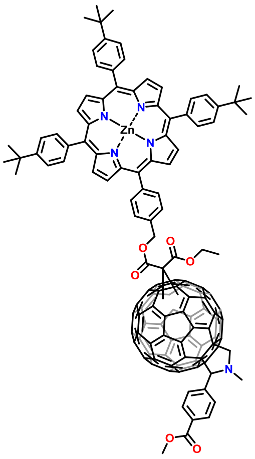

(1) A solution of **8** (1 eqv., 0.0862 g, 0.087 mmol) in 150 mL of DCM was mixed with the solution of **9** (1.5 eqv., 0.12 g, 0.13 mmol) in toluene. Subsequently, CBr_4 (1.1 eqv., 0.0318 g, 0.096 mmol), and after 30 minutes - DBU (1.2 eqv., 0.016 mL, 0.104 mmol) were added. 3 hours later the reaction mixture was concentrated under reduced pressure. The obtained solid was dissolved in carbon disulfide and poured on top of the silica gel column. Unreacted fullerene was washed out from the column with carbon disulfide. Elution with CS_2:$CHCl_3$=1:1 (v/v), CS_2:CH_2Cl_2=1:1 (v/v), CH_2Cl_2 and CH_2Cl_2:MeOH=99:1 (v/v), resulted in elution of bisadduct (mixture of isomers). Yield: 29%. ^{1}H NMR (600 MHz, $CDCl_3$): δ 1.61 (s, 27H), 2.54 (s, 3H), 3.45-3.46 (m, 2H), 3.86 (s, 3H), 3.95 (s, 2H), 4.45-4.49 (m, 5H), 5.69 (s, 1H), 7.54-7.66 (d, *J*=7.5 Hz, 2H), 7.72-7.76 (m, 8H), 8.09-8.15 (m, 8H), 8.71-8.72 (d, *J*=10.7 Hz, 2H), 8.74-8.75 (d, *J*=10.7 Hz, 2H), 8.78-8.79 (d, *J*=6.5 Hz, 2H), 8.87-8.88 (m, 4H). ^{13}C NMR (150 MHz, $CDCl_3$): δ 166.88, 155.90, 152.85, 152.66, 147.16, 146.17, 146.04, 145.78, 145.36, 145.23, 144.22, 142.38, 141.99, 141.88, 140.41, 136.89, 136.41, 135.84, 135.50, 134.35, 130.39, 129.97, 129.38, 123.53, 52.17, 40.01, 38.86, 34.96, 31.80, 30.59, 29.75, 29.01, 23.86, 23.03, 14.06. IR (cm^{-1}): 2951, 2926, 2896 (Bn–H stretching); 1745, 1723 (C=O stretching); 1607 (C=C stretching); 1360 (C–N deformation); 1223, 1202 (C–O stretching); 1062 (C–N stretching); 965 (Zn–N); 795, 716 (C–H deformation). MALDI-TOF MS (m/z): calculated 1901.50 [M^+], found 1901.82 [M^+].

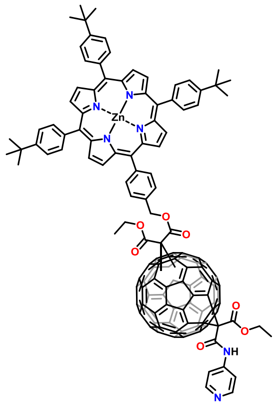

(2) **8** (1 eqv., 0.0882 g, 0.09 mmol) and **11** (1.2 eqv., 0.0996 g, 0.11 mmol) were dissolved in around 150 ml of DCM. Subsequently, CBr_4 (1.1 eqv., 0.0325 g, 0.1 mmol), and after 30 minutes - DBU (1.2 eqv., 0.02 mL, 0.11 mmol) were added. 3 hours later the reaction mixture was concentrated under reduced pressure. The obtained solid was dissolved in carbon disulfide and poured on top of the silica gel column. Unreacted fullerene was washed out from the column with carbon disulfide. Elution with CS_2:$CHCl_3$=1:1 (v/v), CS_2:CH_2Cl_2=1:1 (v/v), CH_2Cl_2 and CH_2Cl_2:MeOH=99:1 (v/v), resulted in elution of bisadduct (mixture of isomers). Yield: 15%. ^{1}H NMR (600 MHz, $CDCl_3$): δ 1.51 (s, 3H), 1.61 (s, 27H), 1.48-1.50 (t, 3H), 3.59 (s, 2H), 4.26-4.30 (m, 2H), 4.60-4.63 (m, 2H), 7.69-7.70 (d, J=6.1 Hz, 2H), 7.72-7.75 (m, 8H), 8.13-8.14 (m, 8H), 8.63-8.65 (d, J=7.2 Hz, 2H), 8.89-8.90 (m, 4H), 8.96-8.97 (m, 4H), 8.81 (s, 1H). ^{13}C NMR (150 MHz, $CDCl_3$): δ 166.70, 150.55, 150.04, 146.63, 145.29, 143.25, 142.05, 140.13, 139.86, 134.68, 134.36, 132.23, 132.15, 132.10, 131.62, 126.37, 123.51, 121.53, 121.39, 120.10, 67.30, 61.73, 41.88, 34.95, 31.78, 29.75, 14.21. IR (cm^{-1}): 2926, 2857, 2801 (Bn–H stretching); 1735, 1722 (C=O stretching); 1646 (C=C stretching); 1362 (C–N deformation); 1269, 1200 (C–O stretching); 1073 (C–N stretching); 992 (Zn–N); 795, 722 (C–H deformation). MALDI-TOF MS (m/z): calculated 1916.48 [M^+], found 1916.57 [M^+].

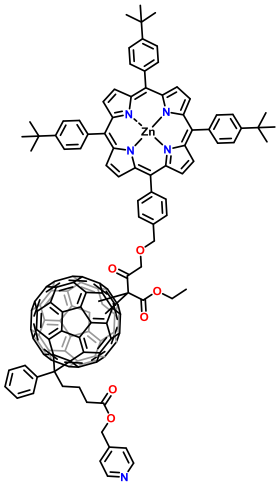

(3) **8** (1 eqv., 0.0743 g, 0.08 mmol) and **14** (1.2 eqv., 0.09 g, 0.09 mmol) were dissolved in around 150 ml of DCM. Subsequently, CBr_4 (1.1 eqv., 0.0275 g, 0.08 mmol), and after 30 minutes - DBU (1.2 eqv., 0.015 mL, 0.09 mmol) were added. 3 hours later the reaction mixture was concentrated under reduced pressure. The obtained solid was dissolved in carbon disulfide and poured on top of the silica gel column. Unreacted fullerene was washed out from the column with carbon disulfide. Elution with CS_2:$CHCl_3$=1:1 (v/v), CS_2:CH_2Cl_2=1:1 (v/v), CH_2Cl_2 and CH_2Cl_2:MeOH=99:1 (v/v), resulted in elution of bisadduct (mixture of isomers). Yield: 27%. ^{1}H NMR (600 MHz, $CDCl_3$): δ 1.24 (s, 27H), 1.48-1.50 (t, 2H), 2.28-2.31 (t, 2H), 2.43-2.45 (t, 2H), 2.56-2.59 (t, 2H), 3.03-3.06 (t, 2H), 3.64 (s, 3H), 3.67 (s, 4H), 6.97-7.00 (m, 5H), 7.42-7.44 (m, 14H), 7.68-7.70 (m, 4H), 7.95-7.96 (m, 10H). ^{13}C NMR (150 MHz, $CDCl_3$): δ 166.53, 150.44, 150.03, 143.40, 134.60, 132.15, 126.56, 121.37, 67.44, 66.85, 56.07, 47.67, 34.89, 32.01 31.73, 29.34, 24.83, 24.30, 14.21. IR (cm^{-1}): 2953, 2920, 2857 (Bn–H stretching); 1737 (C=O stretching); 1607 (C=C stretching); 1332 (C–N deformation); 1202 (C–O stretching); 1067 (C–N deformation); 996 (Zn–N); 799, 718 (C–H deformation). MALDI-TOF MS (m/z): calculated 1977.53 [M^+], found 1977.97 [M^+].

(4) 4-formylbenzoic acid (1 eqv., 1.0 g, 6.66 mmol), 5 mL of hydrochloric acid solution (35 wt. %) were dissolved in 50 mL of MeOH. The flask was connected with the condenser and heated at 65ºC. The reaction was stopped by the addition of an aqueous solution of sodium bicarbonate. The reaction mixture was extracted with ethyl acetate. The organic phase was collected and dried over sodium sulphate. The crude mixture was concentrated under reduced pressure and

purified using flash chromatography (chloroform-hexane gradient elution). Eluent: DCM. Yield: 15%. ^{1}H NMR (600 MHz, $CDCl_3$): δ 1.18 (s, 4H), 7.41-7.48 (m, 2H), 7.79 (d, *J*=8.3 Hz, 2H).

(**5**) Pyrrole (4 eqv., 0.19 mL, 2.8 mmol), methyl 4-formylbenzoate (1 eqv.,, 0.1150 g, 0.70 mmol), 4-*tert*-butylbenzaldehyde (3 eqv., 0.35 mL, 2.1 mmol) were dissolved in 0.6 L of $CHCl_3$. The resulting mixture was deaerated with nitrogen gas for 30 min. The flask was shielded from light and TFA (1.2 eqv., 0.06 mL, 0.84 mmol) was added. After 24 hours DDQ (2 eqv., 0.0318 g; 1.4 mmol) was added and the reaction mixture was left stirring for the next 24 hours. The reaction was stopped by neutralizing with TEA. The reaction mixture was concentrated under reduced pressure and purified using silica gel column. Eluent: chloroform. Yield: 13%. ^{1}H NMR (600 MHz, $CDCl_3$): δ 1.62 (s, 27H), 4.12 (s, 3H), 7.25 (s, 2H), 7.76 (dd, *J*=10.3 Hz, 2H), 8.17-8.15 (m, 6H), 8.33 (d, *J*=8.2 Hz , 2H), 8.45 (d, *J*=7.6 Hz, 2H), 8.79 (d, *J*=4.2 Hz, 2H), 8.91-8.93 (m, 6H). IR (cm^{-1}): 3313 (N–H stretching); 2951, 2900, 2863 (Bn–H stretching); 1723 (C=O stretching); 1604 (C=C stretching); 1358 (C–N deformation); 1186 (C–O stretching); 1098 (C–N stretching); 801, 726 (C–H deformation). MALDI-TOF MS (m/z): calculated 840.44 [M^+], found 841.02 [MH^+].

(**6**) Free base porphyrin **5** (1 eqv., 0.37 g, 0.44 mmol) was dissolved in 300 mL of chloroform, while zinc acetate dihydrate (10 eqv., 0,97g, 4.4 mmol) was dissolved in 10 mL of MeOH. Both solutions were placed in a flat bottom flask and left under stirring for 2 hours. The reaction was stopped by the addition of an aqueous solution of sodium bicarbonate. The organic phase was collected and dried over sodium sulphate. The crude mixture was concentrated under reduced pressure and purified using silica gel column. Eluent: DCM. Yield: 96%. ^{1}H NMR (600 MHz, $CDCl_3$): δ 1.63 (s, 27H), 4.13 (s, 3H), 7.26 (s, 2H), 7.77 (d, *J*=10.3 Hz, 2H), 8.16-8.17 (m, 6H), 8.34 (d, *J*=8,2 Hz, 2H), 8.46 (d, *J*=7.6 Hz, 2H), 8.81 (d, *J*=4.1 Hz, 2H), 8.92-8.93 (m, 6H). IR (cm^{-1}): 2951, 2900, 2863 (Bn–H stretching); 1723 (C=O stretching); 1604 (C=C stretching); 1358 (C–N deformation); 1186 (C–O stretching); 1098 (C–N stretching); 998 (Zn–N); 801, 726 (C–H deformation). MALDI-TOF MS (m/z): calculated 902.35 [M^+], found 902.73 [M^+].

(7): **6** (1.0 eqv., 0.4192 g, 0.46 mmol) was dissolved in 100 mL of dry THF. $LiAlH_4$ was added in portions until no progress of the reaction was observed. Afterwards, few mL of water were added. The reaction mixture was filtrated through the celite and the solvent removed under reduced pressure. The obtained solid was dissolved in dichloromethane and purified using silica gel column. Eluent: DCM. Yield: 91%. ^{1}H NMR (600 MHz, $CDCl_3$): δ 1.61 (s, 24H), 3.51-3.53 (t, 8H), 5.02-4.97 (m, 3H), 7.68 (d, *J*=8.3 Hz, 1H), 7.74 (d, *J*=8.3 Hz, 5H), 8.13-8.14 (dd, *J*=10.3 Hz, 5H), 8.20 (d, *J*=7.6 Hz, 1H), 8.90 (d, *J*=4.8 Hz, 1H), 8.96-8.97 (m, 5H). IR (cm^{-1}): 3024 (O–H stretching); 2957, 2898, 2859 (Bn–H stretching); 1605 (C=C stretching); 1360 (C–N, def.); 1200 (C–O stretching); 1064 (C–N stretching); 998 (Zn–N); 811, 718 (C–H deformation). MALDI-TOF MS (m/z): calculated 874.36 [M^+], found 875.01 [M^+].

(8) **7** (1.0 eqv., 0.1140 g, 0.13 mmol) was dissolved in 125 mL of dichloromethane. Afterwards, 10 mL of dry THF were added. When the clear solution was created, TEA (1.2 eqv., 0.02 mL,

0.16 mmol) was added. After half an hour malonyl chloride (1.2 eqv., 0.02 mL, 0.16 mmol) was added dropwise. The reaction was stopped after 15 hours by the addition of an aqueous solution of sodium bicarbonate. The reaction mixture was extracted three times with dichloromethane. The organic phase was collected and dried over sodium sulphate. The crude mixture was concentrated under reduced pressure and purified using silica gel column. Eluent: DCM. Yield: 65%. ^{1}H NMR (600 MHz, $CDCl_3$): δ 1.32-1.35 (t, 4H), 1.51 (s, 7H), 1.61 (s, 25H), 7.75-7.72 (m, 8H), 8.13 (dd, *J*=9.6 Hz, 5H), 8.22 (d, *J*=8.3 Hz, 3H), 8.89 (d, *J*=4.8 Hz, 3H), 8.97-8.98 (m, 5H). IR (cm^{-1}): 2957, 2902, 2861 (Bn–H stretching); 1739, 1707 (C=O stretching); 1605 (C=C stretching); 1360 (C–N stretching); 1265, 1200 (C– stretching); 1064 (C–N stretching); 996 (Zn–N); 811, 718 (C–H deformation). MALDI-TOF MS (m/z): calculated 988.39 [M^+], found 988.71 [M^+].

(9) Fullerene C_{60} (1.5 eqv., 300 mg, 0.42 mmol), sarcosine (10 eqv., 0.2495 g, 0.8 mmol), (**4**) (1 eqv., 0.046 g, 0.28 mmol) were dissolved in 200 mL of chlorobenzene. The resulting reagent mixture was heated at reflux (130ºC) under argon for 24 hours. After this time, the heating source was removed, and the reaction mixture was cooled to room temperature and the solvent was evaporated under reduced pressure. The obtained residue was dissolved in carbon disulfide and poured on top of the silica gel column. Unreacted fullerene was washed out from the column with carbon disulfide. Elution with CS_2:$CHCl_3$=1:1 (v/v) and $CHCl_3$ resulted in elution of monoadduct. Yield: 42%. ^{1}H NMR (600 MHz, $CDCl_3$) δ (ppm): δ 1.53 (s, 6H), 2.80 (s, 2H), 3.90 (s, 1H), 7.70 (d, *J*=8.9 Hz, 2H), 8.09 (d, *J*=8.3 Hz, 2H). IR (cm^{-1}): 2942, 2920, 2853 (Bn–H stretching); 1718 (C=O stretching); 1607 (C=C stretching); 1329 (C–N deformation); 1273, 1171 (C–O stretching); 1069 (C–N stretching); 736, 718 (C–H deformation). MALDI-TOF MS (m/z): calculated 911.09 [M^+], found 911.03 [M^+].

(**10**) 4-(aminomethyl)pyridine (1 eqv., 0.47 ml, 4.62 mmol) was dissolved in 50 mL of

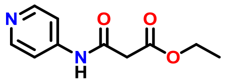

dichloromethane. Subsequently, TEA (1.2 eqv., 0.77 ml, 5.54 mmol) was added. After 30 minutes ethyl malonyl chloride (1.2 eqv., 0.71 ml, 5.54 mmol) was added dropwise. The reaction was stopped after 5 hours by the addition of an aqueous solution of sodium bicarbonate. The reaction mixture was extracted three times with dichloromethane. The organic phase was collected and dried over sodium sulphate. The crude mixture was concentrated under reduced pressure and purified using flash chromatography with a gradient elution (ethyl acetate and hexane). Yield: 71%. ^{1}H NMR (600 MHz, $CDCl_3$): δ 1.15-1.25 (t, 3H), 3.53 (s, 2H), 4.05-4.02 (m, 2H), 8.35-8.45 (d, *J*=13.2 Hz, 2H), 8.55-8.65 (d, *J*=13.0 Hz, 2H), 9.80 (s, 1H).

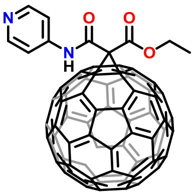

(11) A solution of **10** (1 eqv., 0.1 g, 0.48 mmol) in 10 ml of dichloromethane was added to the solution of C_{60} (1.2 eqv., 0.415 g, 0.58 mmol) in 300 mL of toluene. Subsequently CBr_4 (1.1 eqv., 0.176 g, 0.53 mmol) was added, and after 30 minutes - DBU (1.1 eqv., 0.08 mL, 0.5 mmol). 3 hours later the reaction mixture was concentrated under reduced pressure. The obtained solid was dissolved in carbon disulfide and poured on top of the silica gel column. Unreacted fullerene was washed out from the column with carbon disulfide. Elution with CS_2:$CHCl_3$=1:1 (v/v), CS_2:CH_2Cl_2=1:1 (v/v), CH_2Cl_2 and CH_2Cl_2:MeOH=99:1 (v/v), resulted in elution of bisadduct (mixture of isomers). Yield: 16%. ^{1}H NMR (600 MHz, $CDCl_3$): δ 1.10-1.25 (t, 3H), 4.10-4.25 (m, 2H), 8.35-8.45

(d, J=13.2 Hz, 2H), 8.55-8.65 (d, J=13.0 Hz, 2H), 9.92 (s, 1H). MALDI-TOF MS (m/z): calculated 926.07 [M^+], found 926.72 [M^+].

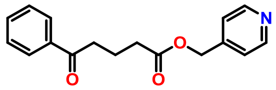

(12) 5-Oxo-5-phenylvaleric acid (1 eqv., 300 mg, 7.28 mmol), 4-pyridinemethanol (1 eqv., 1 g, 9.16 mmol), DMAP (0.02 eqv., 0.022 g, 0.18 mmol) were dissolved in 400 ml dichloromethane. After 30 minutes DCCD (0.87 eqv., 0.5 g, 7.3 mmol) was added. The crude mixture was concentrated under reduced pressure and purified using flash chromatography (ethyl acetate-hexane gradient elution). Yield: 63%.^{1}H NMR (600 MHz, $CDCl_3$): δ 1.94-2.20 (m, 2H), 2.32-2.40 (t, 2H), 2.89-3.00 (t, 2H), 5.98 (s, 2H), 7.45-7.65 (m, 4H), 7.69-7.75 (m, 1H), 7.83-7.95 (dd, J=7.2 Hz, 2H), 8.59-8.62 (d, J=7.3 Hz, 2H).

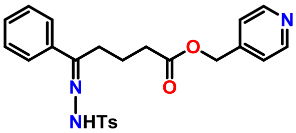

(**13**) (1.0 eqv., 1 g, 4.85 mmol), *p*-toluenesulfonyl hydrazide (1.2 eqv., 1.0839 g, 5.82 mmol) were dissolved in 50 mL of ethanol. The flask was connected with a condenser and heated at 65°C for 18 hours. The crude mixture was concentrated under reduced pressure. The obtained solid was dissolved in dichloromethane and washed with water. The organic phase was collected and dried over sodium sulphate. The crude mixture was concentrated under reduced pressure and purified using flash chromatography (ethyl acetate-hexane gradient elution). Yield: 76%. ^{1}H NMR (600 MHz, $CDCl_3$): δ 2.05-2.19 (m, 4H), 2.27-2.35 (m, 2H), 2.43 (s, 3H), 5.98 (s, 2H), 7.35-7.59 (m, 7H), 7.22-7.30 (d, J=7.5 Hz, 2H), 7.90-8.00 (dd, J=7.0 Hz, 2H), 8.62-8.70 (d, J=7.5 Hz, 2H), 10.50 (s, 1H).

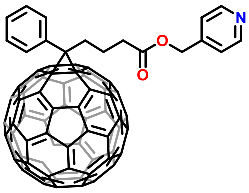

(**14**) (1 eqv., 0.3 g, 0.66 mmol), sodium methoxide (3 eqv., 0.071 g, 1.32 mmol), pyridine (3 eqv., 0.13 mL, 1.32 mmol) and C_{60} (1 eqv., 0.317 g, 0.44 mmol) were dissolved in 150 mL of *o*-DCB. The flask was connected with a condenser and heated at 180°C for 24 hours. After this time, the heating source was removed, and the reaction mixture was cooled to room temperature and the solvent was evaporated under reduced pressure. The obtained residue was dissolved in carbon disulfide and poured on top of the silica gel column. Unreacted fullerene was washed out from the column with carbon disulfide. Elution with CS_2:$CHCl_3$=1:1 (v/v), CS_2:CH_2Cl_2=1:1 (v/v), CH_2Cl_2 and 1% MeOH in CH_2Cl_2 resulted in elution of monoadduct. Yield: 27%. ^{1}H NMR (600 MHz, $CDCl_3$): δ 1.55-1.70 (m, 4H), 2.10-2.20 (t, 2H), 2.25-2.35 (t, 2H), 5.98 (s, 2H), 7.15-7.35 (m, 5H), 7.45-7.50 (d, J=8.2 Hz, 2H), 8.50-8.60 (d, J=10.2 Hz, 2H). IR (cm^{-1}): 2918, 2847 (Bn–H stretching); 1733 (C=O stretching); 1597 (C=C stretching); 1277, 1218 (C–O stretching); 756 (C–H, def.). MALDI-TOF MS (m/z): calculated 988.13 [M^+], found 988.68 [M^+].

3. **Powdered X-ray diffraction**

The powder X-ray diffraction data were measured at 293 K using a SuperNova diffractometer (Agilent) with a CCD detector and a Cu-Kα radiation source at the 150 mm sample-to-detector distance. Fig. 5 presents PXRD images of the as-synthesized titania and the sample annealed at 450°C.

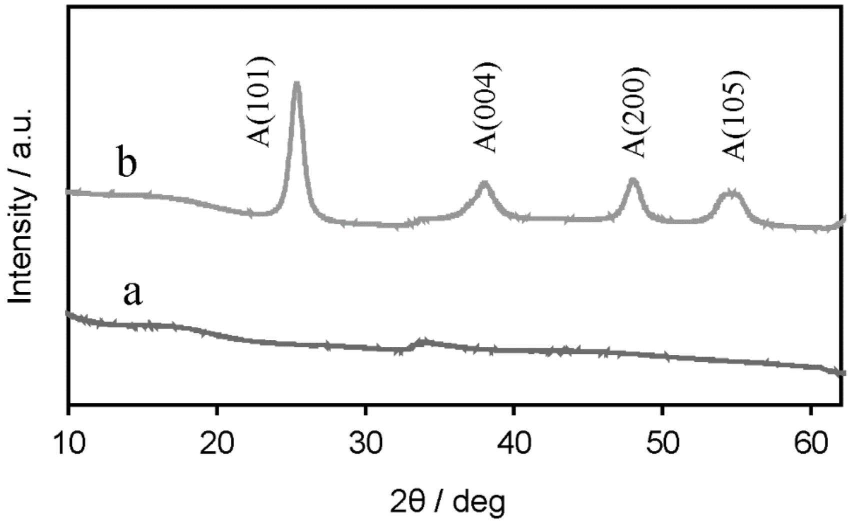

Figure S5. PXRD images of the as-synthesized titania (a) and the annealed at 450°C (b).

4. LC-MS measurements

4.1. PhOH photodegradation intermediates identification conditions:

The ESI source operated in negative ionization mode with the capillary voltage at 4 kV and the sampling cone at 40 V. The source temperature and desolvation temperature were set to 90°C and 250°C, respectively. Data were acquired from 90 to 400 m/z with a 0.5 s scan time. Chromatographic separation of PhOH degradation intermediates was performed on a Kinetex C18 column (2.1 mm × 150 mm, 2.6 μm particle diameter), that was maintained at 25°C. The isocratic elution was performed at a flow rate of 0.2 mL min^{-1}. The mobile phase consisted of acetonitrile and water (40:60, v/v).

Table S1. List of identified compounds obtained in the photocatalytic degradation of PhOH.

Photodegradation product		m/z
1.	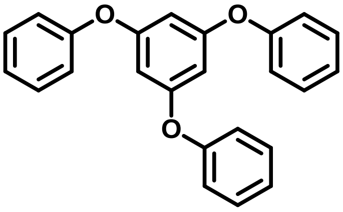	369.11
2.	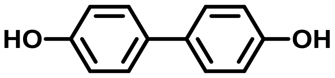	185.06
3.	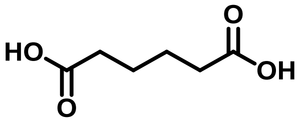	145.06
4.	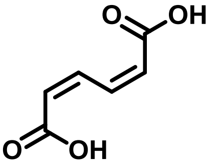	141.03
5.	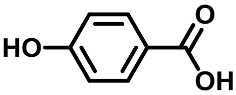	137.03
6.	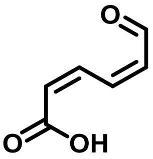	125.03
7.	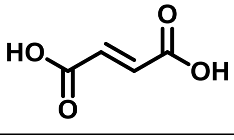	115.00
8.	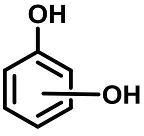	109.03

9.	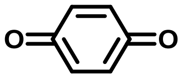	107.01
10.	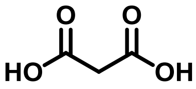	103.01
11.	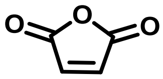	96.99
12.	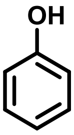	93.03
13.	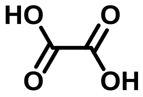	89.00

4.2.MB photodegradation intermediates identification conditions:

The ESI source operated in negative ionization mode with the capillary voltage at 3.5 kV and the sampling cone at 40 V. The source temperature and desolvation temperature were set to 90°C and 250°C, respectively. Data were acquired from 100 to 400 m/z with a 0.5 s scan time. Chromatographic separation was performed on a Kinetex C18 column (2.1 mm × 150 mm, 1.7 μm particle diameter), that was maintained at 25°C. The gradient elution was performed at a flow rate of 0.25 mL min^{-1}. The mobile phase consisted of 0.1% aqueous solution of formic acid (A) and acetonitrile (B). The gradient was changed from 10% B (0min) to 90% B (6 min) and the initial ratio of A:B was 70:30 (v/v). The gradient was as follows: 0 min, 70% A; 15 min, 70% A; 25 min, 20% A; 30 min, 20% A; 35 min, 70% A; 40 min, 70% A.

Table S2. List of identified compounds obtained in the photocatalytic degradation of MB.

Photodegradation product		m/z
1.	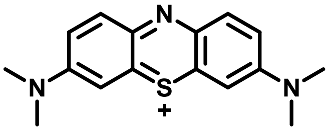	284.40
2.	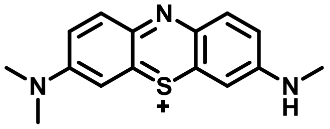	270.37
3.	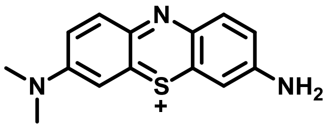	268.36
4.	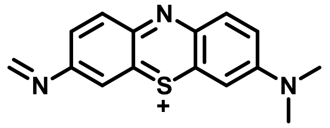	256.35
5.	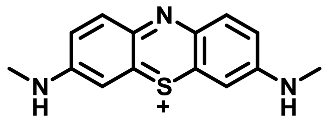	256.35
6.	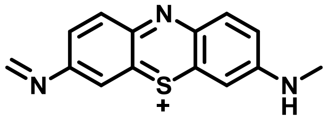	254.33
7.	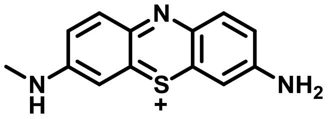	242.32
8.	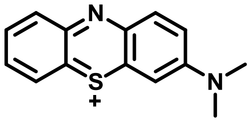	241.33
9.	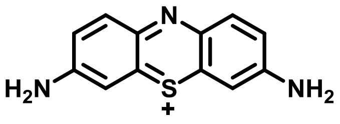	228.29
